# A Numerical Simulation Method for the One-Step Compression-Stamping Process of Continuous Fiber Reinforced Thermoplastic Composites

**DOI:** 10.3390/polym13193237

**Published:** 2021-09-24

**Authors:** Lu Chen, Tianzhengxiong Deng, Helezi Zhou, Zhigao Huang, Xiongqi Peng, Huamin Zhou

**Affiliations:** 1 State Key Laboratory of Materials Processing and Die and Mould Technology, School of Materials Science and Engineering, Huazhong University of Science and Technology, Wuhan 430074, China; d201577253@hust.edu.cn (L.C.); uezuuezu@hust.edu.cn (T.D.); helezizhou@hust.edu.cn (H.Z.); hmzhou@hust.edu.cn (H.Z.); 2 School of Materials Science and Engineering, Shanghai Jiao Tong University, Shanghai 200030, China; xqpeng@sjtu.edu.cn

**Keywords:** CFRTP, compression molding, stamping forming, FEM, ALE, FSI

## Abstract

Continuous fiber reinforced thermoplastic (CFRTP) composites have many advantages, such as high strength, high stiffness, shorter cycle, time and enabling the part consolidation of structural components. However, the mass production of the CFRTP parts is still challenging in industry and simulations can be used to better understand internal molding mechanisms. This paper proposes a three-dimensional simulation method for a one-step compression-stamping process which can conduct thermoplastic compression molding and continuous fiber reinforced thermoplastic composite stamping forming in one single mold, simultaneously. To overcome the strongly coupled non-isothermal moving boundary between the polymer and the composites, arbitrary Lagrangian–Eulerian based Navier–Stokes equations were applied to solve the thermoplastic compression, and a fiber rotation based objective stress rate model was used to solve for the composite stamping. Meanwhile, a strongly coupled fluid structure interaction framework with dual mesh technology is proposed to address the non-isothermal moving boundary issue between the polymer and the composites. This simulation method was compared against the experimental results to verify its accuracy. The polymer flow fronts were measured at different molding stages and the error between simulation and experiment was within 3.5%. The final composites’ in-plane deformation error was less than 2.5%. The experiment shows that this work can accurately simulate the actual molding process.

## 1. Introduction

Continuous fiber reinforced thermoplastic (CFRTP) composites have drawn increasing attention in the automotive industry in recent years because of their peculiar properties such as low weight, high stiffness, and high strength, which make them an ideal material to replace the usage of steel [1]. The thermoplastic matrix of CFRTP also requires a much shorter cycle time than thermoset composites, hence they are highly applicable to the mass production. 

Conventionally the CFRTP plate is heated and placed into the mold to go through automated processes such as matched die molding, rubber pressing, deep drawing, and hydroforming. However, this forming process is limited to relatively simple shell-like structures but is unable produce complex features such as holes, ribs, or bosses, without which the parts cannot be assembled or the structural performance is compromised. To overcome the limitations of the conventional forming process, the injection over-molding process was developed for CFRTP [2]. This is a two-step process: the CFRTP is formed with the normal forming process first; then the preform is placed into the injection mold and extra features are injected over the preform. Such process can produce complex parts with high structural performance, and can be used for large series production in an automated process. This two-step process can be done with either the insert overmolding approach or the two-shot overmolding approach. In the insert overmolding approach, there are two separate molds. The CFRTP is first formed in the forming mold, then it is transferred, either by robots or by hand, into the injection mold as an insert for further injection. In the two-shot overmolding approach, the forming mold and injection mold are integrated, hence this approach is more automated than the insert overmolding approach. The CFRTP is first formed inside the forming cavity, then the rotating platen rotates 180 degrees and brings the formed CFRTP into the other cavity for further injection. Recently the one-step process was developed to further improve the production efficiency. Differently from the two-step overmolding process which conducts the forming and injection sequentially, the one-step process conducts the thermoplastic injection/compression molding and CFRTP stamping forming in one single mold, simultaneously [3].

**Figure 1:**
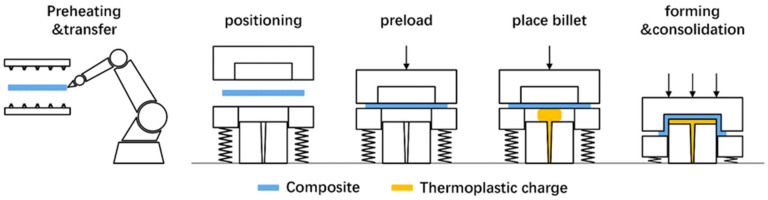
Concept of the one-step compression-stamping process.

Figure 1 illustrates the one-step compression-stamping process. It mainly includes the following operations: (1) preheating and transfer: the CFRTP composites is first heated above its melting temperature, then it is transferred into the mold and constrained by the blank holder. (2) Initial charging: the mold begins to close partly to form the designated cavity size, and then the initial compression charge is placed into the mold. (3) Forming: the mold is closed completely to form the final shape. The compression charge deforms to fill the cavity when the punch moves down, and causes the concurrent CFRTP composites’ deformation as well.

Thermoplastic compression molding is capable of creating complex geometries on top of the CFRTP, hence the formability of CFRTP can be greatly improved. Another advantage of this method is that it requires only one step to obtain the final part, hence this process can be highly automated and is applicable to the automotive industry for mass production. 

A one-step process introduces extra complexities, causing difficulties in production and even failures. Simulation is a powerful tool that can be used for a first-time-right design strategy. Simulation enables the optimization of the design and the molding conditions, predicting potential defects with much lower cost at early stages. In the past decades there various simulation techniques were developed for thermoplastic polymer molding processes. Hieber and Shen [4] adopted the Hele–Shaw model which neglects the inertia and velocity components of the thickness direction for polymer melt flow in thin cavities. Zhou and Li [5] extended the Hele–Shaw model from the mid-plane model to the surface model, which avoids the time-consuming work of constructing the mid-plane. The Hele–Shaw model cannot accurately simulate flow behaviors on chunky geometries, nor the fountain flow effect at the flow front, hence quite a few full three-dimensional simulation methods have been developed. Yan, Zhou, and Li [6] successfully used the three-dimensional finite element method, while Liang [7] applied the three-dimensional finite volume approach. Methods for simulating three-dimensional injection-compression were investigated in the works of Zhang et al [8], Bay and Tucker III [9], Wang and O’Gara [10], and Shaayegan [11], and Li and Zhang [12] investigated fiber orientation behavior of fiber reinforced thermoplastic composites in injection molding.

However, none of the existing methods can be applied directly to this one-step process due to several unresolved difficulties: (1) the fiber models [9,10,11,12] are not suitable for CFRTP composite forming. These models focus on the short and long fibers, which are dispersed inside the polymer with much shorter lengths than CFRTP composites. (2) The process requires modelling the coupled moving boundary physics between the compression charge and the CFRTP composites, but all these existing simulation methods are either based on the fixed computational domain such as conventional injection molding, or a rigid moving boundary such as in compression molding. The shape of the compression charge cannot be predetermined due to the deformation of CFRTP, and a strongly coupled fluid structure interaction is critical to simulating the deformation mechanism accurately. (3) Because of the large thickness variation, a fully three-dimensional mesh is required, but mainstream forming simulation methods use shell elements to avoid the locking problem. Heterogeneous meshes make the simulations converge slowly, especially for this non-isothermal problem when a temperature simulation must also be performed. (4) The computational domain needs to be updated because of the moving interface between the compression charge and the CFRTP composites, hence the arbitrary Lagrangian–Eulerian (ALE) description of motion is needed, and the mesh needs to be deformed. This often leads to distorted finite elements which causes failure in the simulation. Although remeshing can remove the distorted elements, its computation cost is too high for this problem.

The objective of this work is to develop a numerical simulation method for this new process. First, the assumptions and mathematical models are given in Section 2. In Section 3, we discuss a hybrid fluid structure interaction model to solve the non-isothermal moving boundary between the compression charge and CFRTP composites. Section 4 discusses the numerical implementation, especially for the unified temperature solution between the compression charge and the CFRTP composites. In Section 5 and Section 6, we compare the simulation results against the experimental results, and the comparison shows that the simulation matches the experiment with adequate accuracy.

## 2. Mathematics Model

### 2.1. Basic Assumption

This one-step compression stamping molding process can be regarded as the simultaneous deformation of the compression charge and CFRTP composites. The compression charge behaves as a fluid, but the composites behave as solid, hence the assumptions and simplifications are given to the compression charge and CFRTP composites separately.

For the compression charge:

(1) Non-isothermal process. The impact of temperature to the charge is unneglectable, as the viscosity, density, and thermal conductivity are all temperature dependent.
(2) Non-Newtonian fluid. The CROSS-WLF viscosity model is used to describe the viscosity of the thermoplastic charge.
(3) Compressible fluid due to the density is a function of temperature and pressure.

For the CFRTP composites:

(1) Semi-isothermal process. The deformation of the CFRTP composites can be regarded as isothermal process, as the fiber dominates deformation as long as the matrix is above the melting temperature. However, the temperature of the CFRTP composites is important to the compression charge through the contact region, and hence the temperature of the CFRTP composites needs to be solved.

### 2.2. Mathematical Model for Compression Charge Deformation

Continuum mechanics usually use two classical approaches to describe motion: the Lagrangian description, and the Eulerian description. In the Lagrangian description, each mesh node follows the associated material particle during motion, which allows an easy tracking of interfaces, but the large mesh distortion leads to the failure of the numerical code. In the Eulerian description, the thermoplastic charge moves inside the fixed Eulerian domain, hence the description is capable of handling a large distortion. The punch and composites move inside the Eulerian domain, which must be tracked as solid boundaries; however, immerse boundary tracking is not as accurate as the Lagrangian method.

**Figure 2:**
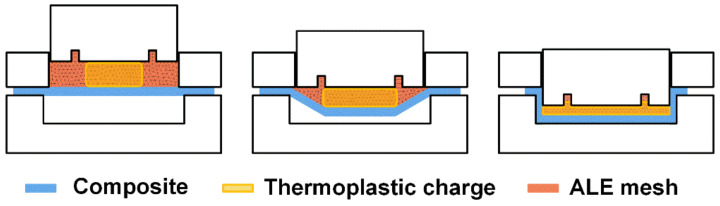
The ALE description for compression-stamping process.

Because of the shortcomings of Lagrangian and Eulerian descriptions, the arbitrary Lagrangian–Eulerian (ALE) description is adopted in this work, which combines the advantages of both the Lagrangian and the Eulerian approaches. In the ALE description, the computational domain is selected as the orange region in Figure 2 and the charge marked as yellow flows inside it, hence capable of handling the large charge distortion; At the mean time the mesh deforms to conform the moving boundaries of the punch and composites which gives the capability of tracking the interfaces accurately [13].

The governing equations based on the ALE description are [13,14]:

(1) $$\frac{\partial \rho }{\partial t}+c\cdot \nabla \rho =-\rho \nabla \cdot v$$

(2) $$\;\rho \left(\frac{\partial v}{\partial t}+\left(c\cdot \nabla \right)v\right)=-\nabla P+\nabla \cdot \tau +\rho g$$

(3) $$\;\rho C_{p}\left(\frac{\partial T}{\partial t}+\left(c\cdot \nabla \right)T\right)=\nabla \cdot \left(k\nabla T\right)+\eta {\dot{\gamma }}^{2}+\beta T\frac{DP}{Dt}$$

In Equations (1)–(3) above, ρ is the density, t is the time, v is the material velocity vector, c is the convective velocity vector, P is the pressure, τ denotes the deviatoric stress tensor, g is the gravitational acceleration vector, $C_{p}$ is the specific heat, T is the temperature, k is the thermal conductivity, η is the viscosity, $\dot{\gamma }$ is the shear rate, and β is the compressibility.

### 2.3. Mathematical Model for CFRTP Composite Deformation

The commonly used CFRTP composites are unidirectional tape and woven fabric with either glass or carbon fibers. The woven fabric composites are more suitable for stamping forming processes, as it is easier to maintain the fabric structure under high temperature. In this work, woven composites are used.

CFRTP composite forming is a large deformation problem, which is usually applicable to the updated Lagrange framework with a hypo-elastic material model. The governing equation of the updated Lagrange method is given by Equation (4) [15].

(4) ∫δD:σdV=∫δv·ρbdV+∫δv·tdS

In Equation (4), σ is the Cauchy stress tensor, D is the rate of deformation tensor, ρ is the material density, b the is body force, and t is the external surface traction. 

**Figure 3:**
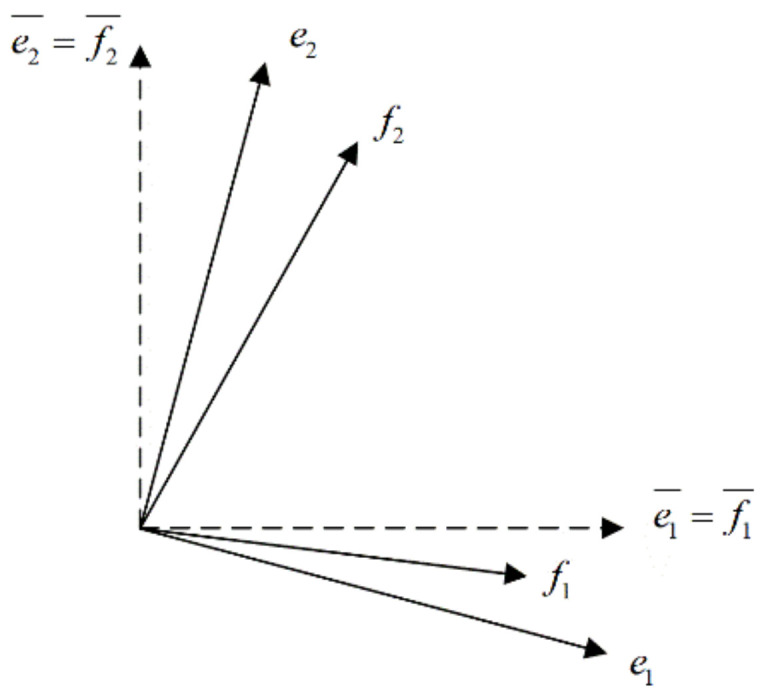
Rigid body rotation and fiber directions.

The equilibrium equation in Equation (4) is still lacking a constitutive model to link the Cauchy stress to the rate of deformation. For path dependent materials, it is more common for the constitutive equations to appear in rate form rather than the stress itself. The Green–Naghdi rate is widely used in finite element codes at finite strains, however it cannot be used for CFRTP composite forming. Because the woven composites are anisotropic materials due to the high stiffness of the fibers, the stress update must be done in the two fiber directions. The standard objective stress rates are based on the average rotation of the continuum which is suitable for the isotropic material. Figure 3 demonstrates the difference between the average material rigid body rotation and the fiber directions. There are two fiber families, $\bar{f_{1}}$ and $\bar{f_{2}}$, which are orthotropic to each other and coincident with the initial basis ($\bar{e_{1}}$, $\bar{e_{2}}$). After deformation, the basis becomes ($e_{1}$, $e_{2}$) under the rigid body rotation effect, which is still orthotropic. However, the current fiber directions $f_{1}$ and $f_{2}$ are no longer orthotropic nor coincident with the basis ($e_{1}$, $e_{2}$). This proves the Green–Naghdi rate cannot be used for updating the fiber stresses, and the stresses must therefore be updated in the fiber directions.

In this paper, we adopted a fiber rotation-based objective stress rate [16] $\sigma^{\nabla \Phi }$ to solve CFRTP composite stamping forming, in which Φ is the rotation tensor of fiber.

(5) $$\sigma^{\nabla \Phi }=\Phi \cdot \left(\frac{d}{dt}\left(\Phi^{T}\cdot \sigma \cdot \Phi \right)\right)\cdot \Phi^{T}$$

### 2.4. Boundary Condition

The Dirichlet boundary condition is applied on the compression charge, which is defined in Equation (6). The velocity boundary condition at the punch is set as the punch speed $v_{m}$, and at the charge and composite contact region the velocity boundary condition is $v_{c}$.

(6) $$v=v_{m},\;\mathrm{at}\;\mathrm{the}\;\mathrm{punch}\;v=v_{c},\;\mathrm{at}\;\mathrm{the}\;\mathrm{charge}-\mathrm{composites}\;\mathrm{interface}$$

The Neumann boundary condition is applied to the CFRTP composites, which is defined in Equation (7). The pressure boundary condition $P_{c}$ is applied to the composites.


(7) $$P=P_{c},\;\mathrm{at}\;\mathrm{the}\;\mathrm{charge}-\mathrm{composites}\;\mathrm{interface}$$

In Equations (6) and (7), $v_{c}$ and $P_{c}$ are unknown. The guessed values are used at the beginning of the analysis, then Equations (1)–(3) and (4) are solved iteratively with the new $v_{c}$ and $P_{c}$ until convergence is reached.

### 2.5. Simulation Algorithm

The complete FSI workflow at time k is given as Table 1.

## 3. Technologies in Dealing with the Moving Boundary

To simulate the complete compression-stamping process, the strong coupling effect at the interface between the compression charge and the CFRTP composites needs to be addressed. From a simulation perspective, this is a strongly coupled fluid structure interaction (FSI) problem because the composite laminate is not rigid. There are two main approaches for the simulation of the fluid structure interaction problem: the monolithic approach and the partitioned approach. According to the work of Shvarts et al. [17], in the monolithic approach the equations governing the charge and the composites are solved within a single solver. This method is accurate and fast as it does not require iterations to reach the interface convergence. However, it requires both fluids and solids to use the same numeric method, which is not the case in this work. According to Küttler and Wall [18], the partitioned approach allows the fluids and solids to be solved in different codebases and meshes, but the cost is calling the fluid solver and solid solver iteratively until the interface convergence has reached. The partitioned approach is slower than monolithic approach and might introduce convergence issues. In this one-step compression-stamping process, both the deformation interface and thermal interface between the compression charge and the CFRTP composites need to be solved, which requires two nested partitioned approaches, and makes the problem much more complicated.

In this work, we used a hybrid approach to solve this non-isothermal moving boundary problem. The partitioned approach was used for the deformation interface, while the monolithic approach was used for the thermal interface. This treatment is superior to either monolithic approach or partitioned approach. To implement the hybrid approach, this paper proposed a dual mesh technique.

### 3.1. Dual Mesh Technique

**Figure 4:**
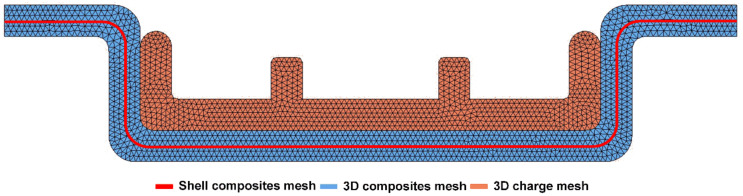
The dual mesh method of composites.

The dual-mesh technique meshes the CFRTP composites into both a shell mesh and three-dimensional tetrahedral mesh. The shell mesh was used for solving the composite deformation, while the three-dimensional tetrahedral mesh was used to solve for the contact and temperature, as the compression charge also uses a three-dimensional tetrahedral mesh. The mapping was established between the shell mesh and three-dimensional mesh, and hence the data can be transferred between each other. In the dual mesh method, the shell elements can be regarded as the middle plane of the three-dimensional tetrahedral elements, as shown in Figure 4.

With the dual mesh treatment, the contact between the three-dimensional composite mesh and three-dimensional charge mesh can be tracked accurately, as the three-dimensional mesh has the full geometric information through the thickness direction. The temperature of the composites and the charge can be solved in a unified thermal solver on the three-dimensional meshes. This is a more efficient approach, as we can apply the heat flux constraint at the charge–composite interface into the energy equation directly, and hence avoid the iterative temperature solution between the composites and the charge. The heat flux constraint at the charge–composite interface is given in Equation (8) [19].

(8) $$q=h\;*\;s\;*\;\left(T_{charge}-T_{composites}\right)$$

In Equation (8), q is the heat through the contact interface, h is the heat transfer coefficient between the charge and composites, s is the contact area, $T_{charge}$ is the charge temperature, and $T_{composites}$ is the composite temperature. When integrating the system matrix for the energy equation, Constraint (8) is applied directly into the system matrix to avoid the temperature iterations between the composites and charge. This treatment allows a one-step temperature solution which is efficient without any convergence issues.

**Figure 5:**
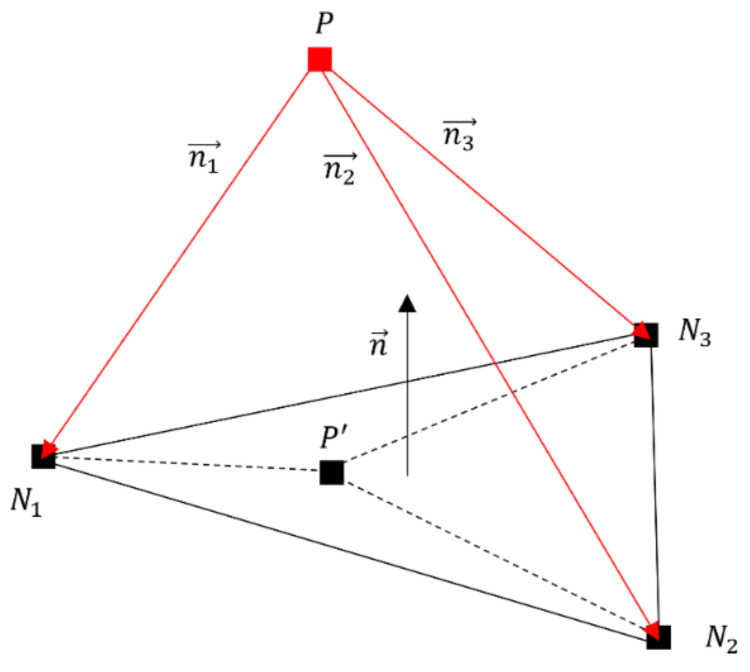
Project node P onto shell element.

Mapping between the three-dimensional tetrahedral elements and the shell elements for CFRTP composites can be done through the procedure below, as shown in Figure 5:

(1) Project the node P of a three-dimensional tetrahedral element onto the shell elements, and denote the projection point as $P^{\prime }$.
(2) Collect all the candidate shell elements which have the projection point $P^{\prime }$ inside.
(3) Find the shell element $S_{min}$ from the candidate shell elements in step 2 which has the minimal distance, and store the local coordinate of $P^{\prime }$ in $S_{min}$.
(4) Determine the location of node P with respect to $S_{min}$. If $\vec{n_{1}}\cdot \vec{n}<0$ then node p is on the positive side. Otherwise, it is on the negative side.
(5) The map from node P to shell element $S_{min}$ is established, and the local coordinate can be used as the interpolation weight. Denote the map as $P\leftarrow S_{min}$.
(6) Add node P into the linked nodes list of $S_{min}$ to build up the map from shell element $S_{min}$ to the three-dimensional tetrahedral nodes. Denote the map as $S\leftarrow \left\{\begin{array}{ccc} P_{1} & \ldots & P_{n} \end{array}\right\}$. From this map, the shell element can link with multiple three-dimensional tetrahedral nodes.

The three-dimensional composite mesh can easily interpolate the pressure field at the contact region. These pressure loads are then mapped from the three-dimensional composite elements to the shell composite elements by the map $S\leftarrow \left\{\begin{array}{ccc} P_{1} & \ldots & P_{n} \end{array}\right\}$ of step 6. The composite deformation code solves the current time step and the composite deformation results are interpolated from the shell elements back to the tetrahedral nodes by the map of $P\leftarrow S_{min}$ of step 5. With the deformation results on the three-dimensional mesh, the ALE method can update the compression mesh accordingly.

### 3.2. Mesh Optimization

Both the three-dimensional compression mesh and the CFRTP composite mesh requires finite mesh deformation. One simple way is to solve the mesh deformation as the elastic problem with fictitious material properties. Let node coordinates x be the unknowns and apply the variational principle on the energy equation (9), with the elastic constitutive model (10) [20]. This equation can be solved by the standard finite element procedure.

(9) $$E_{elastic}\left(x\right)=\frac{1}{2}\int \sigma :\varepsilon dV$$

(10) $$\;\sigma =2\mu \varepsilon +\lambda tr\left(\varepsilon \right)\;I$$

In (9) and (10), σ is the stress tensor,ε is the strain tensor, I is the second order identity tensor, and μ and λ are the Lame constants. μ=1 and λ=1 are used in this work since it is a fictitious material. However, this is insufficient in practice as the solved node coordinates can generated inverted tetrahedral elements, which is unacceptable in numerical code. Many mesh quality improvements and untangling algorithms have been proposed to solve this problem [21,22]. In this work, we introduced an extra penalty term, which was proposed by Liu et al [23], into Equation (9) to ensure optimal elements when deforming the mesh. With the penalty term, the energy function now can be defined by (11),

(11) $$E\left(x,\tau \right)=\frac{1}{2}\int \left(\sigma :\varepsilon +\tau \underset{i=1}{\sum }p\left(\lambda_{i}\left(S\right)\right)\right)dV\;p\left(\lambda \right)={\left(\lambda -\varepsilon \right)}^{2}\;\;\;\mathrm{if}\;\lambda <\varepsilon \;p\left(\lambda \right)=0\;\;\;\;\;\;\mathrm{if}\;\lambda \geq \varepsilon$$

where S is the symmetric part of the deformation gradient, $\lambda_{i}\left(S\right)$ means the i-th eigen value of the tensor S, ε is an infinite small value ($\varepsilon =1.0\times 10^{-6}$ in this work), τ is a relaxation factor which is increased gradually from 1.0 × 10^−6^ to 1.0 × 10^7^ in this work, to ensure the numerical convergence.

## 4. Numerical Implementation

Equations (1) and (2) are coupled in velocity and pressure, which means Equation (1) cannot be solved without taking Equation (2) into account, and vice versa. A computationally efficient method for such a problem is the mixed finite element method [20]. In this work, a mixed velocity-pressure solution is applied by adopting the mini element method proposed by Arnold et al [24].

**Figure 6:**
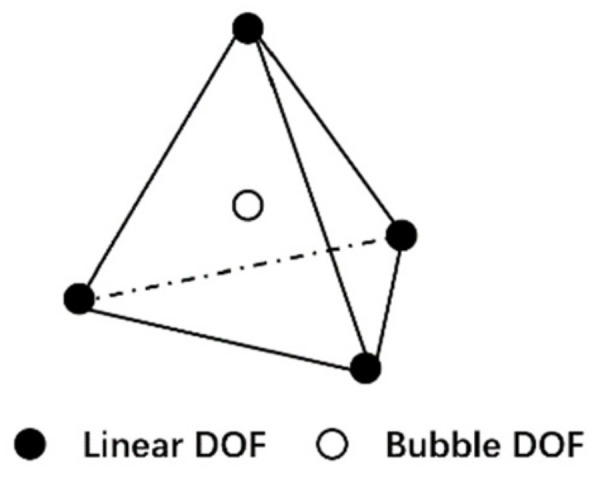
The mini tetrahedral element.

The advantage of the mini element stabilizing technique is that the mini element satisfies the Inf-Sup condition while maintaining the simplicity of linear elements such as the 4-node tetrahedral elements. In the mini element, as shown in Figure 6, one extra bubble velocity degree of freedom is added to the center of the linear element, hence the element velocity is written as follows,

(12) $$v=v_{l}+v_{b}$$

In Equation (12), $v_{l}$ is the linear velocity and $v_{b}$ is the bubble velocity, which is non-zero only inside the element but vanishes on the element boundaries. By using the mini tetrahedral element with a quadratic bubble shape function [25], the standard mixed finite element discretization procedure can be applied Equations (1) and (2).

As the dual-mesh technique is applied in this work, energy equation (3) is actually extended to both the compression charge and the CFRTP composites, but the CFRTP composites are regarded as solid, so only the transient term and diffusion term needs to be solved. The temperature equation for the CFRTP composites is defined in Equation (13),

(13) $$\rho C_{p}\frac{\partial T}{\partial t}=\nabla \cdot \left(k\nabla T\right)$$

The discretization of the energy equation is done via the standard finite element procedure on the linear tetrahedral elements. The whole temperature field can be solved by integrating the compression charge with the Equation (3) domain, and the CFRTP composite domain with Equation (13) separately, then assembling them into one single matrix. Finally, the interface heat flux conservation condition is applied. 

**Figure 7:**
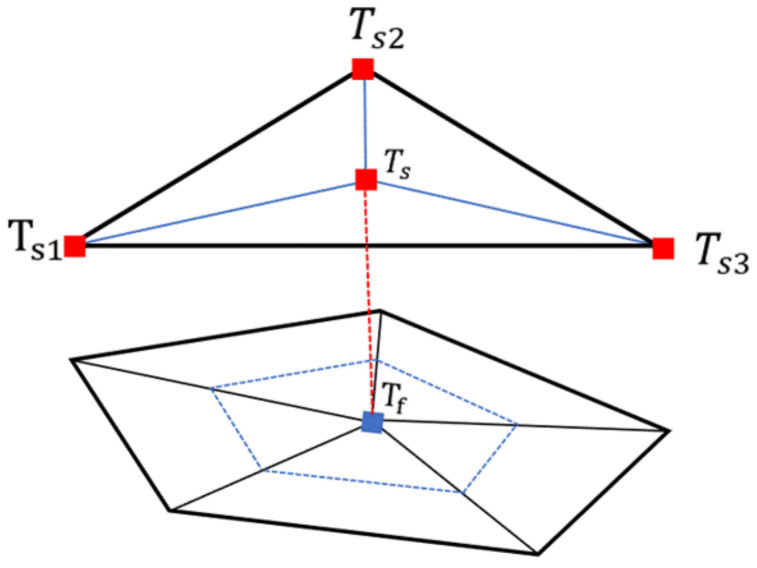
The thermal contact discretization method.

The heat flux constraint in Equation (8) still needs to be implemented. For an arbitrary compression charge node $T_{f}$ on the contact interface, its contact point $T_{s}$ should be inside one of the CFRTP composite elements at the contact interface. Figure 7 demonstrates the contact situation.

From Figure 7, $T_{s}$ can be represented by the element nodal temperatures $T=T_{s1}{\;T}_{s2}{\;T}_{s3}{}^{T}$ and its interpolation function $N=N_{1}\;N_{2}\;N_{3}$.


(14) $$T_{s}=N_{1}T_{s1}+N_{2}T_{s2}+N_{3}T_{s3}$$

Substituting (14) into (8) yields,

$$q=hST_{f}-hS\overset{3}{\underset{i=1}{\sum }}N_{i}T_{si}$$

Equation (15) contains one degree of freedom, $T_{f}$, in the compression charge domain and three degrees of freedom, $T_{si}$, in the CFRTP composites domain. Apply this condition to the corresponding row of the system matrix. Do the same for the CFRTP composite nodes at the contact interface, and then the interface thermal conservation condition is done.

Equation (4) is solved by the explicit finite element method under the updated Lagrange framework in Abaqus, and the constitutive model (5) is implemented inside the user subroutine.

## 5. Experiment and Simulation Setup

The bias-extension test is performed to determine the shear modulus and shear lock angle of the composites constitutive model mentioned in Section 2.3 The test conditions are listed in Table 2.

**Figure 8:**
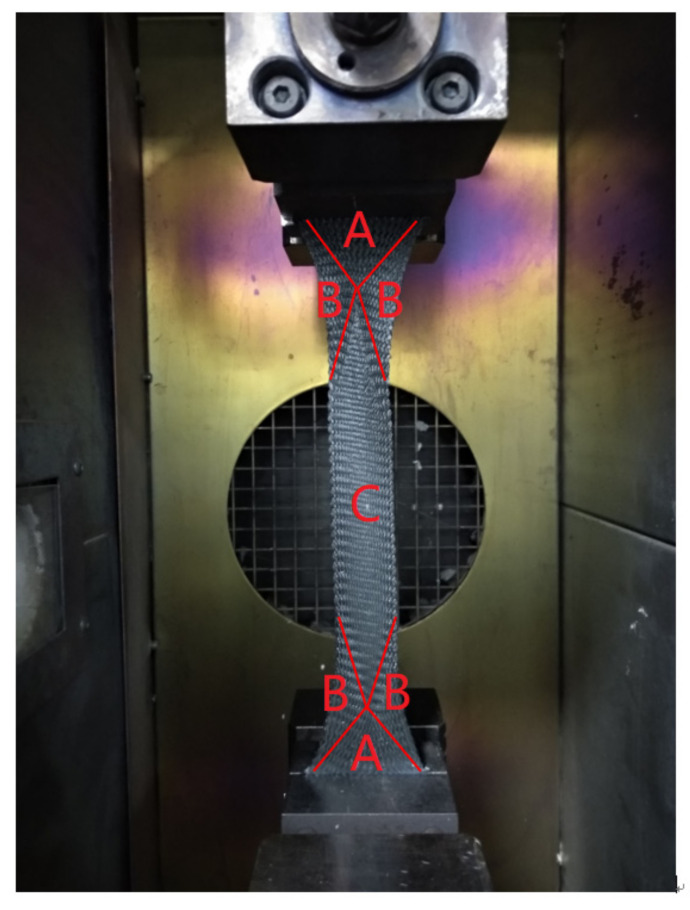
Final state of the bias-extension test.

Figure 8 illustrates the final state of the extension tests, and three regions are shown: A is the fixed region; B is the transition region; and C is the shear region. By using the linear fitting method proposed by Badel, et al. in 2009 [16], we found the shear lock angle was 50 degrees; the shear modulus was 54,000 Pa when the shear angle was less than shear lock angle; and the shear modulus was 1.8 × 10^6^ Pa when the shear angle was greater than the shear lock angle. These composite material properties will be used in the following molding experiment.

**Figure 9:**
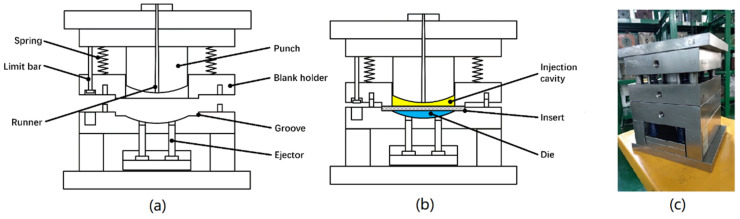
The validation mold: (**a**) open state, (**b**) initial closure, (**c**) mold.

A mold was designed for validating this compression-stamping molding process (shown in Figure 9). The runner is designed with the large gate intentionally to deliver the melt polymer (initial charge) into the cavity with negligible pressure and velocity, so the injection stage of the initial charge can be neglected to simplify the simulation.

In this experiment, the material of the compression charge was polypropylene. The CFRTP composites were made of T300 carbon fiber of 5 layers with a polypropylene thermoplastic matrix, the fiber orientations were 0/90 degrees, and the size of the composites was 140 mm × 140 mm. The molding condition is given in Table 3, below.

## 6. Results and Discussion

### 6.1. Validation of the CFRTP Composite Deformation Model

**Figure 10:**
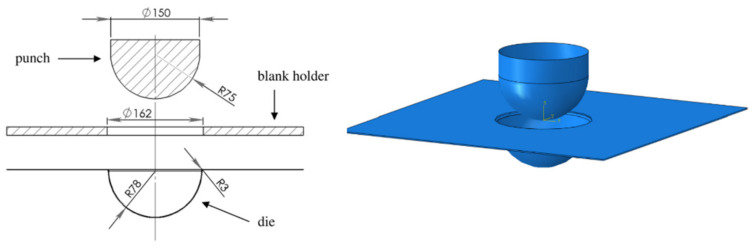
Hemispherical tool design.

The hemisphere simulation in Abaqus was performed based on the composites constitutive model in Section 2.3 to investigate the behavior of the model. The simulation setup and actual experimental results are from the work of Babel [16]. The dimensions of the tool are shown in Figure 10. The Young moduli for both fiber directions were 35,400 MPa, the shear locking angle $\gamma_{limit}$ was set to 50 degrees, and the shear coefficient $G\left(\gamma \right)$ was set to 0.3 MPa if the current shear angle γ was lower than $\gamma_{limit}$, otherwise $G\left(\gamma \right)$ was set to 40 MPa.

**Figure 11:**
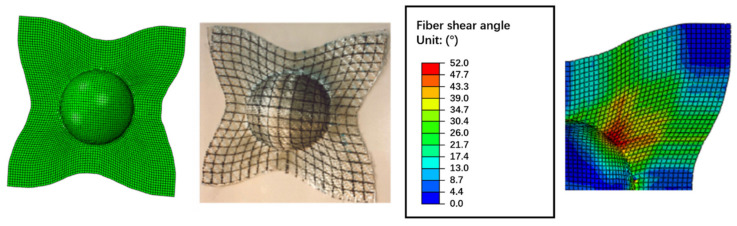
Deformation of the CFRTP composites with 0/90 degree fiber angles.

**Figure 12:**
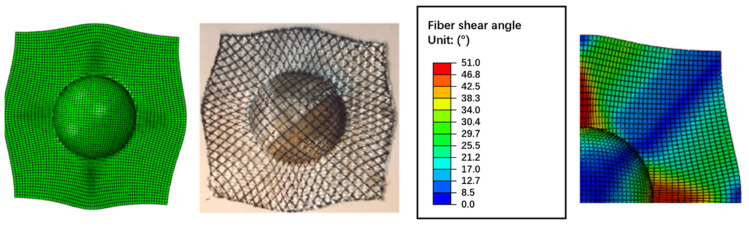
Deformation of the CFRTP composites with -45/45 degree fiber angles.

The initial fiber angles of 0/90 and −45/+45 degrees were tested. Figure 11 and Figure 12 compare the simulated shape against the actual molded shape, and they show a good correlation between numerical and experimental results. It can see that the initial fiber angles play an important role to the final deformation, and the constitutive model can correctly model such behavior. In Figure 11, the maximum shear angle (SDV6, 52 degrees) appears in the −45 and 45 degree directions, while the minimum shear angle appears in the 0 and 90 degree directions. This is the correct behavior as the initial fiber angles are 0/90 degrees, and hence composites in these two directions have a very large extension modulus and the deformation is extension dominated, the maximum shear regions are in the −45 and 45 degree directions. On the contrary, in Figure 12, the initial fiber angles were −45/45 degrees, and hence the maximum shear angle (SDV6, 51 degrees) appears in the 0 and 90 degree directions, as in these regions the deformation is shear dominated.

### 6.2. Results of Applying the Dual Mesh

**Figure 13:**
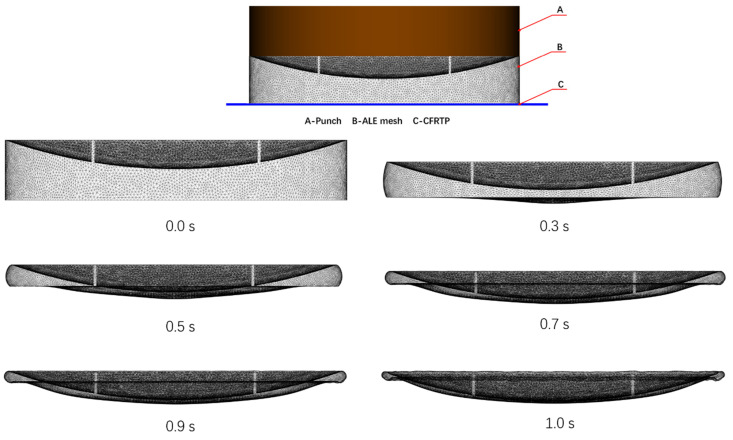
Compression mesh deformation.

Figure 13 shows the ALE mesh setup for the compression charge, and how the mesh deforms during the compression process. It can be seen that the mesh was squashed significantly, and such mesh distortion generated inverted elements which caused numerical solution failures. By applying the mesh optimization technique in Section 3.2, the mesh can fix the inverted elements by minimizing the penalty energy, to guarantee the mesh is still in good condition even after such a large deformation.

According to Equation (11), the penalty energy term is non-zero only when there are inverted elements, and the number of inverted elements is proportional to the value of the penalty energy. This means the value of the penalty energy can be used as an indicator to the quality of the mesh, a smaller penalty energy means better mesh quality. Table 4 compares the penalty energy without and with mesh optimization under the time steps in Figure 11. In order to get the penalty energy without mesh optimization, we simply solve the Equation (11) by setting τ = 0.0. After that, we solve Equation (11) for current time step by increasing τ from 1.0 × 10^−6^ to 1.0 × 10^7^ gradually, and the penalty energy at τ = 1.0 × 10^7^ is used as the value after mesh optimization.

From Table 4, it can be seen that the penalty energy is small even without optimization at the early stages, such as 0.0 s and 0.3 s, this is because the mesh has not deformed significantly yet. As the time progresses and the mesh is squashed to a much smaller thickness, the penalty energy becomes larger if no mesh optimization is applied and usually the FEM solver would fail with errors, but if the mesh optimization is applied to the mesh, we can observe the penalty energy can be minimized, which means the mesh quality was improved. At the very late stages such as 1.0s, the penalty energy without optimization can be 10,000 times larger than the penalty energy with optimization.

**Figure 14:**
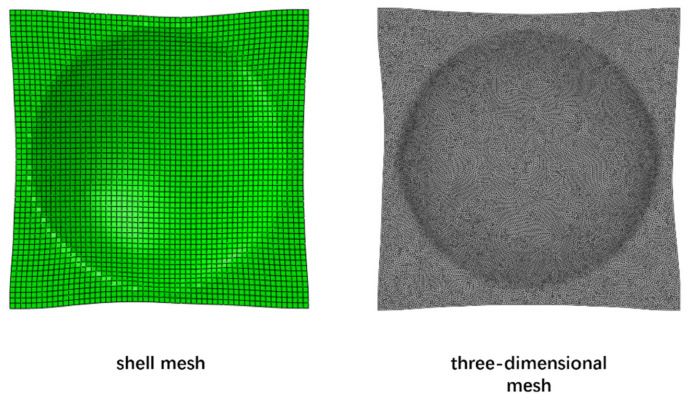
Dual mesh deformation.

The dual-mesh technique meshes the CFRTP composites into both the shell mesh and the three-dimensional tetrahedral mesh. Figure 14 shows the deformation results of the dual mesh, the left side is the shell composite mesh and the right side is the three-dimensional composite mesh. The three-dimensional composite mesh interpolates the deformation results which are actually solved on the shell mesh in Abaqus with the method proposed in Section 3.1. Figure 14 shows that the three-dimensional deformation matches the shell deformation well. Thus, we can use the three-dimensional mesh to handle the contact and solve the temperature. 

**Figure 15:**
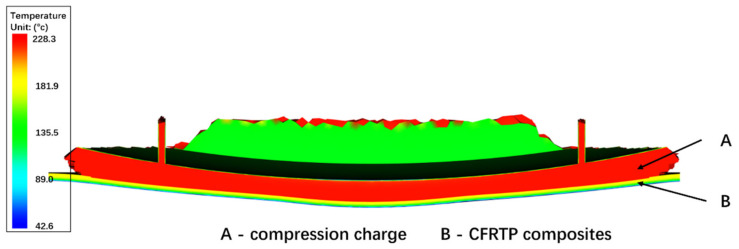
Temperature of compression charge and CFRTP composites at 0.3 s.

Figure 15 shows the temperature field of the compression domain and composite domain at the Y-Z cutting plane. As the compression charge and composites are solved in the unified temperature solution, the heat can conduct through these two domains without any extra numerical iterations. The large temperature gradient of the CFRTP composites through the thickness direction can be observed due to its upper surface being in contact with the hotter compression charge.

**Figure 16:**
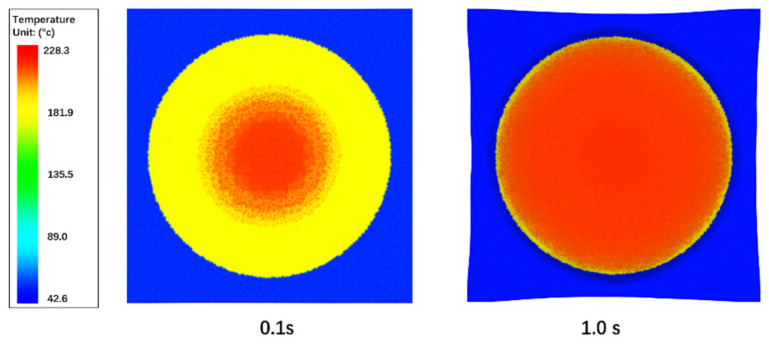
Temperature of the contact surface at 0.1 s and 1.0 s.

Figure 16 gives the more detailed temperature results at the contact regions. It can be seen that the contact surface is divided into 3 regions by temperature. The red region is in contact with the compression charge, and hence has the highest temperatures. The yellow region is in contact with air as the compression charge has not filled this region yet, so the composites in this region remains close to its initial temperature, 180 degrees. The blue region is in contact with the black holder which is set at 40 degrees as the boundary condition, so it is the coldest region. Such temperature results indicate that the heat flux boundary conditions at the contact regions are applied correctly by using the dual mesh technique. Because both the compression charge and the composites use three dimensional meshes for thermal contact, the contact region of the filled region, unfilled region and mold region can be detected easily. At the mean time, the unified thermal system matrix can be integrated over the three-dimensional compression charge and composites meshes without extra thermal iterations between the compression charge and the composites, this guarantees the numerical convergence and accuracy.

### 6.3. Validation of the Melt Flow Front 

The experiment and simulation set the maximum compression displacement as 25 mm and completes the compression in 1 second. To validate the intermediate deformation results, three tests were carried out by stopping the punch at the displacement of 10 mm, 15 mm, and 25 mm (marking D10, D15, D25), which is 0.4 s, 06 s, and 1.0 s in time. This test is similar to the short shot experiment for injection molding. The difference is the amount of compression charges were the same but the compression distances were different in these three runs. This test demonstrates how the part is formed in the actual mold under different stages, and hence can be used to validate the intermediate simulation results. 

**Figure 17:**
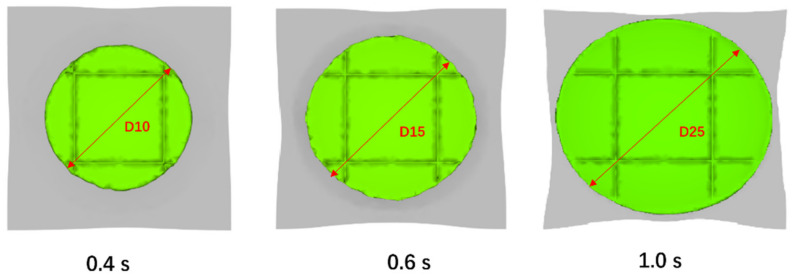
Top views of compression flow front at 0.4 s, 0.6 s and 1.0 s.

**Figure 18:**
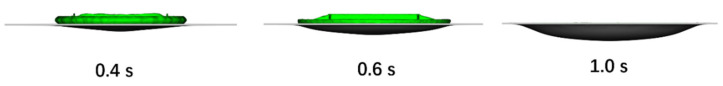
Side views of compression flow front at 0.4 s, 0.6 s, and 1.0 s.

**Figure 19:**
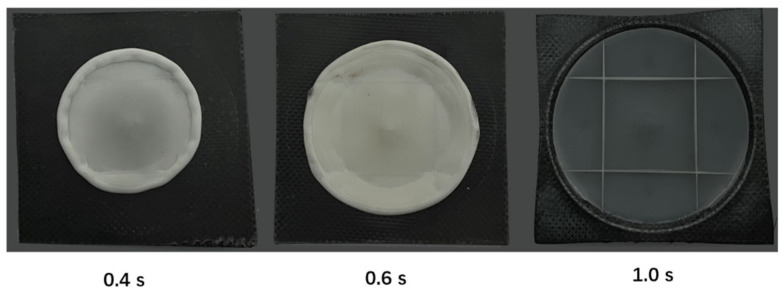
Top views of molded parts at 0.4 s, 0.6 s, and 1.0 s.

Figure 17, Figure 18, Figure 19 show the experimental results and the simulation results at these different compression distances. From these results, it can be seen that with the compression distance increases, the compression charge expands to fill the cavity and the CFRTP composites deform concurrently. As in this model the compression is a disk-like flow pattern and the compression volume and the CFRTP composites are keeping changing, we can use the diameters D10, D15, and D25 marked in Figure 17 to compare the accuracy of the compression. We measured D10, D15, and D25 on the actual molded parts and the simulation, and listed the comparison in Table 5. From the comparison, it can be seen that the simulation results match the experiment well.

**Figure 20:**
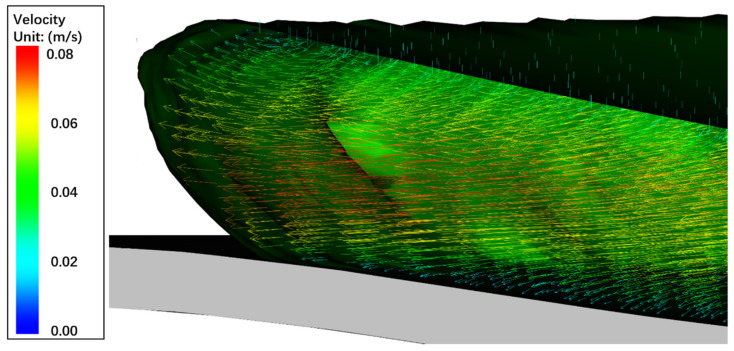
Velocity field of the compression flow front.

Figure 20 illustrates the details of the compression flow front. The gray region is the CFRTP composite, and the green region is the compression. The vectors indicate the velocities field, in which the red vectors stand for higher velocities and blue vectors stand for lower velocities. This is pressure driven flow, and the velocity field plot indicates that the polymer will flow both in the X-Y plane and the Z direction. The full three-dimensional movement forms the final geometry, and the Z direction deformation drives the deformation of the CFTRP composites as well. 

### 6.4. CFRTP Composites In-Plane Deformation

**Figure 21:**
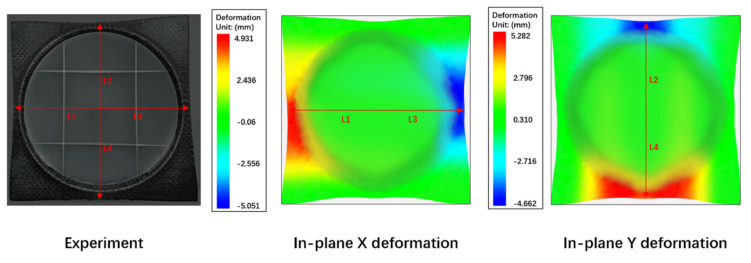
Comparison in-plane deformations between formed products and simulation.

One of the most important simulation results is the CFRTP composite in-plane deformation. In this model, the CFRTP composite deformation is driven by the pressure of the compression charge. The compression charge expands and deforms as the punch moves down, which generates pressure on the composite surface. Such pressure causes the CFRTP composite to deform both in-plane and out-of-plane. The in-plane deformation is controlled by the fiber angle layout. This model uses a 0/90 degree fiber angle layout, so according to the analysis in Section 6.1, the four corners of the composite should remain fixed while the four edges should deform toward the center. Figure 21 shows the final in-plane deformation results of the experiment and the simulation.

The original half edge length of the CFRTP composite plate was 70 mm. As the maximum in-plane deformation happens at the four edge centers, L1, L2, L3, and L4 shown in Figure 21 can be measured to compare the simulation’s accuracy against the experiment. L1 and L3 measure the deformed length in the X direction, while L2 and L4 measure the deformed length in the Y direction. In Figure 21, the in-plane X deformation plot shows that the maximum deformation in the X direction was 4.931mm and the minimum deformation in X direction was −5.051 mm, so the L1 length from the simulation was 65.07 mm and the L3 length was 64.95 mm. Similarly, L2 and L4 can be computed from the in-plane Y deformation plot, and were 65.34 mm and 64.72 mm. Table 6 lists the simulation and experimental values of these four lengths. The maximum error of L1, L2, L3 and L4 between simulation and experiment was 2.44%, and averaged error was 1.53%. This reason why the simulation matches the experiment well is because the constitutive model in Section 2.3 can describe the fiber rotation accurately during the deformation and hence update the stress correctly.

**Figure 22:**
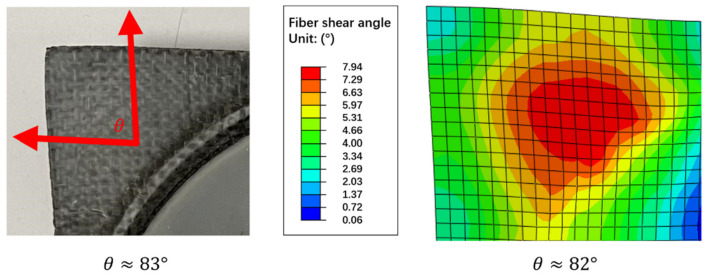
Comparison of fiber angles.

Figure 22 further compares the final shear angle between the molded part and the simulation. The initial shear angle was 0 degrees, and the shear angle increased as the in-plane shear increased. The simulation shows the maximum fiber angle was 7.94 degrees, so angle θ was about 82 degrees. The measured angle θ on the actual molded part was about 83 degrees.

## 7. Conclusions

In this work we developed a numerical algorithm to simulate the one-step compression-stamping forming process, and the major contributions of this work are:

1. We developed a numerical algorithm to simulate this one-step compression-stamping process.
2. We proposed a hybrid fluid structure interaction (FSI) model to solve the moving boundary between the compression charge and the CFRTP composites, and a novel dual-mesh technique was proposed to handle this non-isothermal moving boundary.
3. We developed a concurrent mesh deformation and optimization algorithm to avoid inverted finite elements when deforming the mesh. This algorithm is more efficient than remeshing the whole computational domain.
4. We compared the simulation results against the experimental results to verify the accuracy: the polymer flow fronts error between the simulation and the experiment was within 3.5%. The final composite in-plane deformation error was less than 3%. The experiment shows that this work can accurately simulate the actual molding process.

## Figures and Tables

**Table 1:** Complete FSI workflow.

**Algorithm 1: FSI Workflow**		
Given time = k		
Repeat		
	Repeat	
		Update flow front
		Solve velocity and pressure
		Solve temperature
		Update material properties
	Until compression charge converges	
	Update pressure boundary condition for composites	
	Solve composites deformation	
	Update velocity boundary condition for charge Update the flow mesh	
Until FSI converge		

**Table 2:** The bias-extension test conditions.

Test Conditions	Values
Material	TORAY T300 carbon fiber and polypropylene matrix
Fiber extension modulus	230E9 Pa
Temperature	180 degrees (453.15 K)
Sample width	60 mm
Sample height	150 mm
Sample thickness	0.3 mm
Speed	10 mm/s
Distance	60 mm

**Table 3:** Molding conditions.

Molding Conditions	Values
Charge melt temperature	220 degrees (493.15 K)
Composites pre-heated temperature	180 degrees (453.15 K)
Mold temperature	40 degrees (313.15 K)
Compression distance	25 mm (0.984 in)
Compression speed	25mm/s (0.984 in/s)
Packing time	2 s
Cooling time	50 s
Initial charge weight	25 g (0.055 lb)

**Table 4:** Value of penalty energy.

Time	Penalty Energy without Optimization	Penalty Energy with Optimization
0.0 s	0.0	0.0
0.3 s	$1.38\times 10^{-4}$	0.0
0.5 s	$5.65\times 10^{-2}$	$2.57\times 10^{-8}$
0.7 s	$1.35\times 10^{-1}$	$4.17\times 10^{-8}$
0.9 s	$2.46\times 10^{1}$	$4.71\times 10^{-2}$
1.0 s	$9.14\times 10^{3}$	$2.61\times 10^{-2}$

**Table 5:** Flow front comparison.

Case	Simulation (mm)	Experiment (mm)	Error (%)
D10 (mm)	87.1	90	3.22
D15 (mm)	114.9	118.7	3.20
D25 (mm)	124	120	3.33

**Table 6:** CFRTP composite in-plane deformation error comparison.

Case	Simulation (mm)	Experiment (mm)	Error (%)
L1	65.07	64.1	1.51
L2	65.34	66.3	1.45
L3	64.95	63.4	2.44
L4	64.72	65.2	0.73

## References

[1] Vaidya U., Chawla K. (2008). Processing of fibre reinforced thermoplastic composites. Int. Mater. Rev..

[2] Rietman B., Boxus E., Muhammad K.S., Verghese N. (2016). Manufacturing Solutions for Hybrid Overmolded Thermoplastic UD Composites. http://www.temp.speautomotive.com/SPEA_CD/SPEA2016/pdf/TP/TP12.pdf.

[3] TPRC. https://tprc.nl/news/check-out-our-video-on-automated-one-step-overmolding-of-cpeek.

[4] Hieber C., Shen S. (1978). Flow analysis of the nonisothermal 2-dimensional filling process in injection-molding. Isr. J. Technol..

[5] Zhou H., Li D. (2001). A numerical simulation of the filling stage in injection molding based on a surface model. Adv. Polym. Technol. J. Polym. Process. Inst..

[6] Yan B., Zhou H., Li D. (2007). Numerical simulation of the filling stage for plastic injection moulding based on the Petrov-Galerkin methods. Proc. Inst. Mech. Eng.Part B J. Eng. Manuf..

[7] Liang J., Luo W., Huang Z., Zhou H., Zhang Y., Zhang Y., Fu Y. (2017). A robust finite volume method for three-dimensional filling simulation of plastic injection molding. Eng. Comput..

[8] Zhang Y., Yu W., Liang J., Lang J., Li D. (2018). Three-dimensional numerical simulation for plastic injection-compression molding. Front. Mech. Eng..

[9] Bay R.S., Tucker III C.L. (1992). Fiber orientation in simple injection moldings. Part I: Theory and numerical methods. Polym. Compos..

[10] Wang J., O’Gara J.F., Tucker III C.L. (2008). An objective model for slow orientation kinetics in concentrated fiber suspensions: Theory and rheological evidence. J. Rheol..

[11] Shaayegan V., Ameli A., Wang S., Park C.B. (2016). Experimental observation and modeling of fiber rotation and translation during foam injection molding of polymer composites. Compos. Part A Appl. Sci. Manuf..

[12] Li M., Zhang Y., Zhang S., Hou B., Zhou H. (2020). Experimental investigation and modeling study of the fiber orientation behavior. Eng. Comput..

[13] Donea J., Huerta A., Ponthot J.P., Rodríguez-Ferran A. (2017). Arbitrary Lagrangian–Eulerian Methods. Encycl. Comput. Mech. Second Ed..

[14] Kennedy P., Zheng R. (2013). Governing equations. Flow Analysis of Injection Molds.

[15] Belytschko T., Liu W.K., Moran B., Elkhodary K. (2013). Lagrangian and Eulerian Finite Elements in One Dimension. Nonlinear Finite Elements for Continua and Structures.

[16] Badel P., Gauthier S., Vidal-Sallé E., Boisse P. (2009). Rate constitutive equations for computational analyses of textile composite reinforcement mechanical behaviour during forming. Compos. Part A Appl. Sci. Manuf..

[17] Shvarts A.G., Vignollet J., Yastrebov V.A. (2021). Computational framework for monolithic coupling for thin fluid flow in contact interfaces. Comput. Methods Appl. Mech. Eng..

[18] Küttler U., Wall W.A. (2008). Fixed-point fluid–structure interaction solvers with dynamic relaxation. Comput. Mech..

[19] Lienhard I., John H. (2005). Laminar and turbulent boundary layers. A Heat Transfer Textbook.

[20] Bathe K.-J. (2006). Finite Element Procedures.

[21] Kim J., Panitanarak T., Shontz S.M. (2013). A multiobjective mesh optimization framework for mesh quality improvement and mesh untangling. Int. J. Numer. Methods Eng..

[22] Sastry S.P., Shontz S.M., Vavasis S.A. (2014). A log-barrier method for mesh quality improvement and untangling. Eng. Comput..

[23] Liu T., Gao M., Zhu L., Sifakis E., Kavan L. (2016). Fast and Robust Inversion-Free Shape Manipulation.

[24] Arnold D.N., Brezzi F., Fortin M. (1984). A stable finite element for the Stokes equations. Calcolo.

[25] Pierre R. (1995). Optimal selection of the bubble function in the stabilization of the P1-P1 element for the Stokes problem. SIAM J. Numer. Anal..

